# Quantitative thyroglobulin response to radioactive iodine treatment in predicting radioactive iodine-refractory thyroid cancer with pulmonary metastasis

**DOI:** 10.1371/journal.pone.0179664

**Published:** 2017-07-13

**Authors:** Chen Wang, Xin Zhang, Hui Li, Xin Li, Yansong Lin

**Affiliations:** 1 Department of Nuclear Medicine, Peking Union Medical College Hospital, Beijing, China; 2 Department of Nuclear Medicine, Zhejiang Cancer Hospital, Hangzhou, Zhejiang, China; Advanced Centre for Treatment Research and Education in Cancer, INDIA

## Abstract

**Objective:**

Current diagnosis of radioactive iodine (RAI)-refractory (RAIR) differentiated thyroid cancer (DTC) is based on the imaging technique, which is of a high cost. Serum thyroglobulin (Tg) is a sensitive and easily obtained biomarker. Hence, we aimed to assess the predicting value of quantitative response of Tg in earlier identifying the RAIR-DTC with pulmonary metastasis.

**Patients and methods:**

Pulmonary metastatic DTC patients who underwent total or near-total thyroidectomy and at least two times of RAI therapy were included in this study. The pre-ablative stimulated Tg at the first and second RAI therapy were defined as pstim-Tg1 and pstim-Tg2, while the suppressed Tg before and after the second RAI therapy were designated sup-Tg1 and sup-Tg2. The predicted value of pstim-Tg2/Tg1 and sup-Tg2/Tg1 ratio were detected using the receiver operating characteristic (ROC) curve and logistic regression analyses.

**Results:**

Totally 115 patients were involved in this study. ROC curves showed a cut-off value of 0.544 for pstim-Tg2/ pstim-Tg1 in detecting RAIR, with a sensitivity of 0.9 and specificity of 0.477, and an area under the curve (AUC) of 0.744. Similarly, the cut-off of sup-Tg2/ sup-Tg1 was 0.972, with a sensitivity of 0.733 and specificity of 0.935, and AUC of 0.898. Univariate analysis illustrated that age, tumor size, pstim-Tg2/Tg1, sup-Tg2/ sup-Tg1 and BRAF^V600E^ mutation were eligible to predict RAIR. While from multivariate analysis, only age, pstim-Tg2/Tg1, sup-Tg2/ sup-Tg1 and BRAF^V600E^ mutation were verified to be the independent predictive factors.

**Conclusion:**

The quantitative Tg response was encouraging in identifying RAIR-DTC with pulmonary metastasis. Age, BRAF^V600E^ mutation and Tg response were independent predictors in predicting RAIR-DTC.

## Introduction

These years, with a rapidly rising incidence, thyroid cancer has gained globlal concern. Differentiated thyroid carcinoma (DTC) originates from aberrant follicular cells, accounting for nearly 95% of all thyroid tumors [1]. In general, most DTCs can achieve peculiarly good prognosis under surgery, radioactive iodine (RAI) and thyroxine therapy [2]. Unfortunately, up to 10% of DTC patients develop distant metastases, the most frequent cause of cancer-related death, and 30% of whom become radioiodine-refractory DTC (RAIR-DTC) [3,4]. With a 10-year survival rate less than 10%, RAIR-DTC has received wide attention [4,5]. RAI therapy is an effective way for DTC patients with metastasis. However, patients would benefit little from RAI therapy if they do not respond or become refractory to ^131^I. Hence, recognizing the RAIR-DTC in time is imperative for such patients, with an aim to avoid unnecessary RAI therapy and gain more time to the effective treating regimen like tyrosine kinase inhibitor. Till now, ^18^F-fluorodeoxyglucose positron emission tomography / computed tomography (^18^FDG-PET/CT) is the only recommendation for identifying RAIR-DTC in guidelines, in combination with whole body scan (WBS), CT after multiple RAI therapy and serum thyroglobulin (Tg) [3]. Though convincing, these methods sometimes may be high-cost and time-consuming to prevent those patients from the unbenefited RAI treatment. As a sensitive biomarker, Tg tests are indispensable for DTC patients, due to the usefulness during the follow-up as well as in predicting distant metastasis [2,6,7]. Our previous studies have demonstrated the impressing value of Tg in decision-making and prognosis estimation [8,9,10]. Decreasing of Tg after RAI implies the potential benefit from the therapy, while the increasing or stabilizing of Tg reflects the unsatisfied efficacy, which may be latent to be RAIR-DTC [11]. In 2015 American Thyroid Association (ATA) guideline on the management of thyroid nodule and thyroid cancer, the change of Tg to therapy has been adopted as one of the essential parts in response to therapy system[2], while little study has focused on predictive performance of quantitative Tg response in RAIR-DTC so far. Easily obtained and low-cost, as well as a routine test during the follow-up, is it feasible to use Tg response alone to forecast RAIR as early as just after the second RAI therapy?

In this research, we aim to assess the predictive value of Tg response in identifying RAIR in DTC patients with pulmonary metastasis.

## Patients and methods

This study was deemed to be exempt from the requirement for review by the ethics committee of Peking Union Medical College Hospital. Informed consent was obtained from all individual participants included in the study. All procedures performed in studies involving human participants were in accordance with the ethical standards of the Peking Union Medical College Hospital and with the 1964 Helsinki declaration and its later amendments or comparable ethical standards.

### Patients

This was a retrospective analysis for including DTC patients who underwent total or near-total thyroidectomy and RAI therapy at our hospital from Jan. 2008 to Dec. 2015. Inclusion criteria were as follows: (1) aged 18 years or older, (2) underwent total or near-total thyroidectomy of DTC, (3) present of pulmonary metastasis, (4) underwent at least two times of RAI therapy, (5) data were eligible for analysis, (6) negative thyroglobulin antibody (TgAb). Patients would be defined as pulmonary metastasis with any of the following: (1) pulmonary metastatic lesions confirmed by pathology, (2) focal or diffuse uptake in pulmonary metastatic lesions on whole body scan (WBS) after excluding the contamination and physiological RAI uptake, with or without positive findings on other complementary imaging modalities (chest CT, x-rays, MRI, bone scintigraphy or ^18^F-FDG PET/CT) or elevated Tg levels; (3) positive findings on ^18^F-FDG PET/CT after excluding other malignancies and benign diseases, with a rising Tg level, despite of the negative WBS results; (4) negative findings on functional imaging, but structural lesions suggested by other imaging instruments with a rising Tg level after excluding other malignancies and benign diseases.

### Postoperative RAI therapy and follow-up strategy

A serum thyroid stimulating hormone (TSH) more than 30 mU/L was achieved by thyroid hormone withdrawal (THW) before RAI therapy. All patients were instructed with a low iodine diet from the beginning of THW to 3 weeks after RAI therapy. Patients were administrated with a radioactive iodine dose of 3.7–7.4GBq (100-200mCi) according to the physicians. The second RAI therapy was usually performed after 6 to 12 months after first therapy. A serum stimulated or suppressed thyroglobulin, TSH, thyroglobulin antibody (TgAb) were usually followed 1 day before and 2–3 months after RAI therapy. Stimulated Tg was defined as Tg measured after THW or rhTSH with TSH level >30 mU/L. Post-therapeutic whole-body scan (Rx-WBS) was performed 5–7 days after RAI therapy. High resolution computed tomography (CT) without contrast was carried out according to the patients`situation. All patients were under TSH suppressive therapy by using sodium levothyroxine with TSH < 0.1 μIU/mL. The presence of RAIR was defined as the end-point event, and the non-RAIR duration (survival time) was defined as the time period from the initial diagnosis of thyroid cancer to the end-point event occurrence.

### Measurement of Tg, TgAb and TSH

Tg and TgAb levels were determined by electrochemiluminescence immunoassay (provide by Roche Diagnostics GmbH, Mannheim, Germany) with a functional sensitivity of 0.100 ng/mL and 10 IU/mL, respectively. TSH was determined by chemiluminescence immunoassay (provided by Siemens Healthcare Diagnostics Inc, New York, New York, USA), with a measuring range from 0.004 to 150 μIU/mL. TgAb values >100 IU/mL were considered positive. WBS was obtained in the anterior and posterior projections using dual-head gamma cameras (Infinia Hawkeye; GE, Fairfield, Connecticut, USA) equipped with high-energy parallel-hole collimators, and a 20% energy window was centered at 364 keV, at a table speed of 20 cm/min for a total time of 15 min (256×1024 matrix).

### Radioactive iodine-refractory and radioactive iodine-avid assessment

Till the last time of follow-up, RAIR-DTC was defined if the patient meets either of the followings: (1) patients with distant metastatic disease that does not take up radioactive iodine according to the ^131^I-WBS, (2) had one measurable lesion that had progressed within the past 12 months even it could uptake radioiodine (3) received cumulative activity of ^131^I over 600 mCi. Pulmonary metastases were divided into ^131^I-avid and non-^131^I-avid according to the results of ^131^I-WBS.

### Tg assessment profile

A series of Tg were available during the therapy and follow-up. In this research, we conducted the quantitative analysis of stimulated Tg and suppressed Tg, respectively. The stimulated Tg and TSH at the day of first (pstim-Tg1, TSH1) and second (pstim-Tg2, TSH2) time of RAI therapy, and the suppressed Tg at 1 to 3 months before (sup-Tg1) and 3 months after (sup-Tg2) the second RAI therapy were further analyzed to detect the prediction value.

### BRAF^V600E^ mutation analysis

Genomic DNA was extracted from four 5μm-thick slices of formalin-fixed paraffin-embedded primary tumor samples, using a commercial DNA extraction kit (GeneRead DNA formalin-fixed paraffin-embedded kit, Qiagen, Catalog No. 180134, Germany). Exon 15 of the BRAF gene containing the site for the T1799A (V600E) mutation was PCR amplified using primers 5’-TGCTTGCTCTGATAGGAAAATG-3’ (sense) and 5’-AGCCTCAATTCTTACCATCCA-3’ (antisense). PCR protocol comprised an initial denaturation of 3 min at 95°C, 40 amplification cycles (denaturation for 30 s at 95°C, annealing for 30 s at 60°C and extension for 30 s at 72°C) and final extension for 10 min at 72°C. After quality confirmation by agarose gel electrophoresis, the PCR products were subjected to Sanger sequencing performed using an ABI Prism 3730 DNA Analyzer (Applied Biosystems, Villebon sur Yvette, France).

### Statistical analysis

For univariate analysis, the Mann-Whitney test was used for quantitative and qualitative variables. The Chi-square test and Fisher exact test were used to compare the categorical data. Logistic regression was used in multivariate analysis. A *P* value less than 0.05 was considered significant. Receiver operating characteristic (ROC) curve analysis was used to determine the cut-off level of Tg for predicting the radioactive iodine-refractory. Time to RAIR duration analyses was estimated using the Kaplan-Meier method. The log-rank test was used for comparisons between groups. The multiple Cox model was used to calculate the hazard ratio (HR) between groups. Statistical analysis was carried out using SPSS (version 19.0; SPSS Inc, Chicago, Illinois, USA) and Prism 6 (GraphPad Software, San Diego, CA, USA).

## Result

### Characteristics of the patients

A total of 115 patients were involved in this study for further analysis, 4 of whom were follicular thyroid carcinoma (FTC), 111 of papillary thyroid carcinoma (PTC). The mean age of the involved patients was 43.04 years at the time the first RAI therapy. The ratio of female to male was 2.1:1. The mean follow-up time was 55.17±22.77 months. The mean pre-ablative TSH level was 81.6 and 91.0 μIU/mL at the first and the second RAI therapy, respectively. A total of 50 patients were considered to be RAIR (S1 File), among which, 41 were of non-^131^I-avid WBS after the successful thyroid-ablation, 3 of accumulated RAI dose more than 600 mCi, and 6 of progression disease after high-dose RAI therapy within 12 months. Most patients (92/115) were American Joint Committee on Cancer (AJCC) stage T3 or T4. All 115 patients were all eligible for further stimulated Tg analysis. Besides, 76 patients were available for suppressed Tg analysis. Totally 71 patients meet all the parameters were involved in the logistic regression analysis. The details of the included patients were shown in Table 1.

**Table 1:** The disease-related characteristics of the included patients.

Characteristics	Total N(%)
Sex	
Female	78(67.8)
Male	37(32.2)
Age	
Mean±SD(years)	43.04±14.31
Histologic Type	
Papillary	111(96.5)
Follicular	4(3.5)
Extra-thyroid extension	
Yes	93(80.9)
No	14(12.2)
NA	8(6.9)
AJCC T Stage	
T1	9(7.8)
T2	7(6.1)
T3	39(33.9)
T4	53(46.1)
NA	7(6.1)
AJCC N Stage	
N0	5(4.3)
N1a	23(20.0)
N1b	80(69.6)
N/A	7(6.1)
AJCC M Stage	
M0	0(0.0)
M1	115(100)
Metastasis region	
Pulmonary Only	107 (93.0)
Pulmonary and Bone	8 (7.0)
RAI Therapy dose (mCi)	
First	147±30
Second	150±20
Preablative TSH level (μIU/mL)	
First	81.6±40.9
Second	91.0±39.7
Follow-up time	
Mean±SD(month)	55.2±22.8
RAIR	
Yes	50(43.5)
No	65(56.5)
WBS	
^131^I-avid	74(64.3)
Non-^131^I-avid	41(35.7)
Detection of Non-^131^I-avid Lesions	
CT combined with Tg	33(80.5)
PET combined with Tg	8(19.5)

Abbreviation: TSH: thyroid stimulating hormone; RAI: radioactive iodine; RAIR: radioactive iodine-refractory; WBS: whole body scan

### Tg level in RAIR and non-RAIR patients

**Fig 1:**
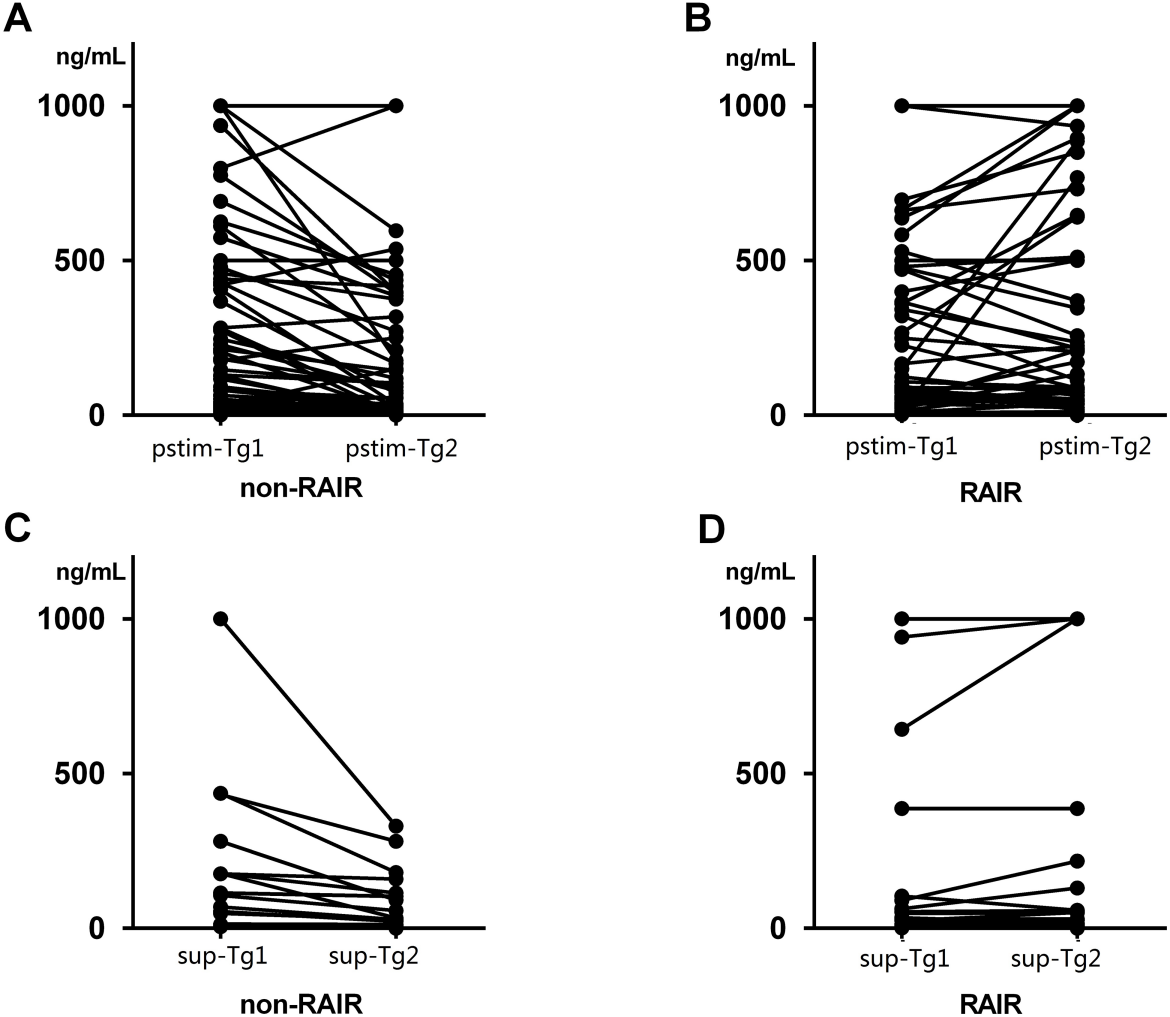
The relevant Tg level of radioactive iodine-refractory (RAIR) and non-RAIR group. A, B: pstim-Tg level at the first and second RAI therapy; C, D: sup-Tg level before and after the second RAI therapy.

The Tg level presented disparity between non-RAIR and RAIR group.The mean pstim-Tg1 level in non-RAIR and RAIR group was 346.1±44.71 and 362.3±49.37, respectively(*P* = 0.56). And mean pstim-Tg2 level of 258.2±42.3 and 415.7±54.18, respectively (*P* = 0.003). Furthermore, the mean sup-Tg1 was 70.74±25.59 and 118.8±48.66 in non-RAIR and RAIR group, respectively (*P* = 0.056). While the mean sup-Tg2 was 33.18±10.65 and 88.76±29.24, respectively (*P*<0.001) (Fig 1). Totally 12.3% (8/65) patients presented increasing pstim-Tg level after the first RAI therapy in non-RAIR group, compared to 30% (15/50) in RAIR group. Similarly, only 2.2% (1/46) cases showed increasing sup-Tg after the second RAI therapy in non-RAIR group, and 63.3% (19/30) in RAIR group.

### ROC curve analysis and diagnostic performances

**Fig 2:**
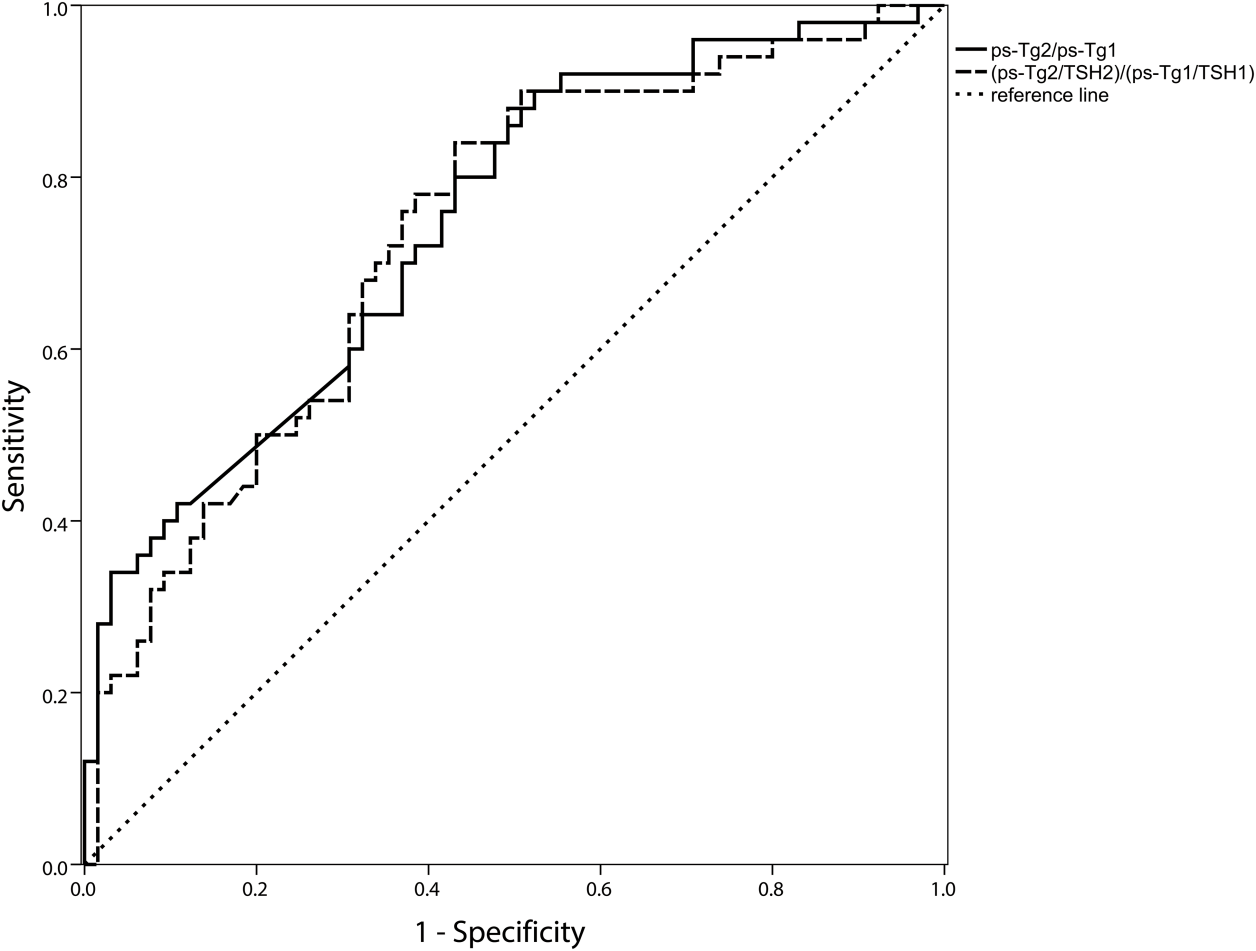
Receiver operating characteristic (ROC) curves of pstim-Tg2/pstim-Tg1 for detecting the radioactive iodine refractory. Area under the ROC curve (AUC): 0.747(95%CI: 0.658–0.836), cut-off value: 0.544, sensitivity: 0.9, specificity: 0.477, negative predict value: 0.861, positive predict value: 0.570.

**Fig 3:**
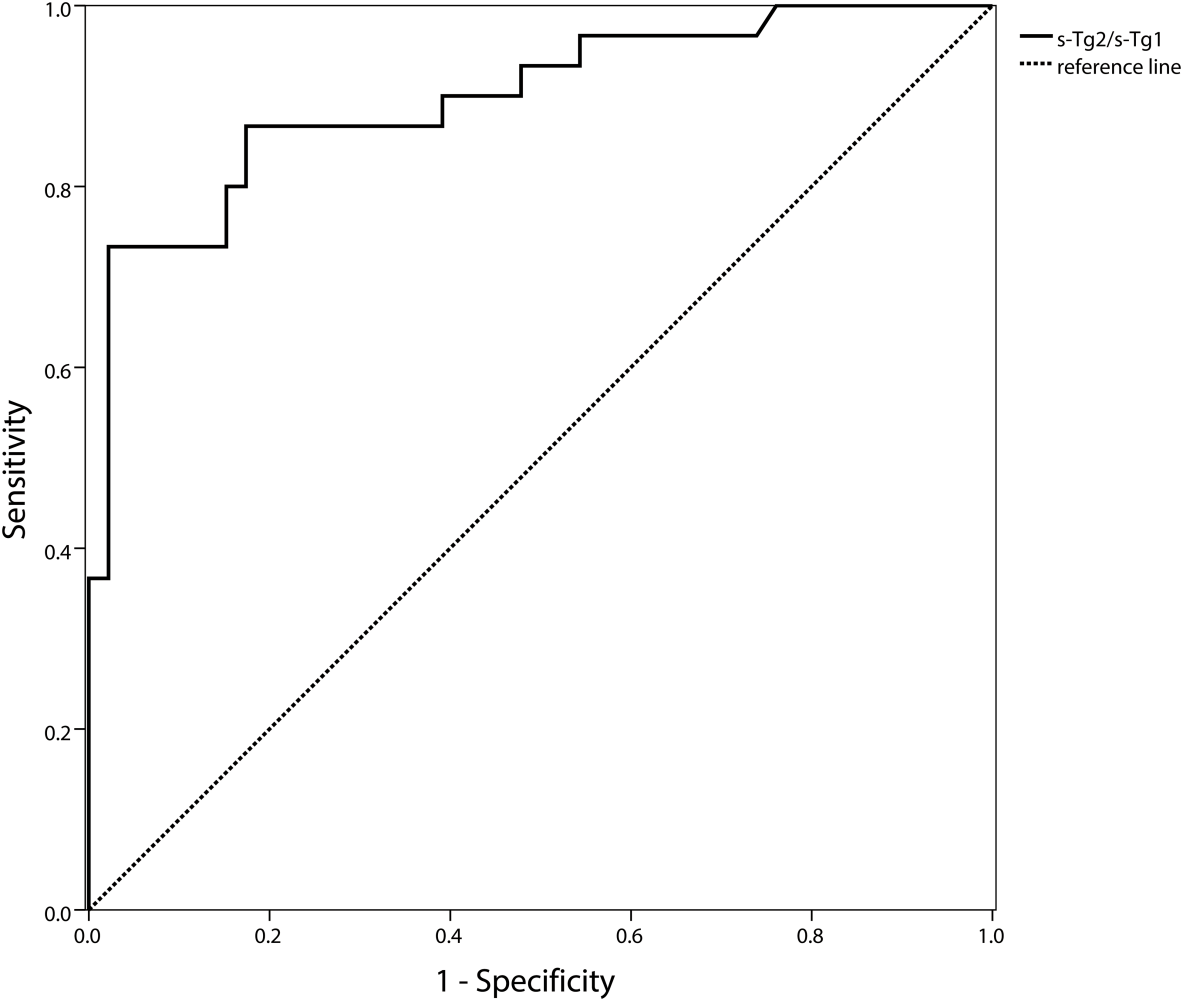
Receiver operating characteristic (ROC) curves of sup-Tg2/sup-Tg1 for detecting the radioactive iodine refractory. Area under the ROC curve (AUC): 0.898(95%CI: 0.823–0.974), cut-off value: 0.972, sensitivity: 0.733, specificity: 0.935, negative predict value: 0.843, positive predict value: 0.88.

We further used ROC curve to detect diagnostic performances and the cut-off value of Tg2/Tg1 in predicting the RAIR. ROC curves showed a fairly good performance of quantitative Tg in detecting RAIR. For stimulated Tg (pstim-Tg), a cut-off value of pstim-Tg2/ pstim-Tg1 at 0.544 was achieved in detecting patients with RAIR, with a sensitivity of 0.9 and specificity of 0.477, and an area under the curve (AUC) of 0.747. To minimize the influence of TSH, (pstim-Tg2/TSH2)/(pstim-Tg1/TSH1) was also performed in the analyses. A cut-off value of (pstim-Tg2/TSH2)/(pstim-Tg1/TSH1) at 0.564 was attained with sensitivity of 0.84, specificity of 0.477, and AUC of 0.734, respectively (Fig 2), indicating a non-superior value of pstim-Tg/TSH than pstim-Tg alone. Then, only pstim-Tg was used for further analysis. Moreover, suppressed Tg showed a higher specificity. Regarding suppressed Tg, a cut-off value of sup-Tg2/sup-Tg1 at 0.972 was obtained, with a sensitivity of 0.733 and specificity of 0.935, and AUC of 0.898, respectively (Fig 3). The higher Tg2/Tg1 ratio above the cut-off value, the more likely to be RAIR-DTC. Combined test represented a sensitivity of 0.9 and specificity of 0.609 in parallel, and 0.733 and 0.935 in series, respectively, which showed no improvement.

### Influence factors and logistic regression analyses

Factors including gender (male or female), age (≥45 years or <45 years), tumor size, tumor type (unifocality or multifocality), extra-thyroidal extension, AJCC T stage, N stage, pstim-Tg2/pstim-Tg1 (≥0.544 or <0.544), sTg2/sTg1 (≥0.972 or <0.972) and BRAF^V600E^ mutation, were analyzed using the univariate and multivariate logistic regression. In univariate logistic regression analyses, the age (odds ratio (OR): 3.65, 95% confidence interval (CI): 1.349–9.876, *P* = 0.011), tumor size (OR:1.439, 95%CI: 1.042–1.988, *P* = 0.027), pstim-Tg2/pstim-Tg1 (OR: 12.745, 95%CI: 3.322–48.897, P<0.001), sup-Tg2/sup-Tg1 (OR: 34.125, 95%CI: 8.174–142.458, *P*<0.001)) and BRAF^V600E^ mutation (OR: 4.091, 95%CI: 1.497–11.18, *P* = 0.006) were eligible to predict the RAIR. In multivariate logistic regression analyses, only the age (OR: 10.484, 95%CI: 1.009–108.942, *P* = 0.049), pstim-Tg2/pstim-Tg1 (OR: 10.829, 95%CI: 1.098–106.789, *P* = 0.041), sTg2/sTg1 (OR: 54.232, 95%CI: 3.614–813.853, *P* = 0.004) and BRAF^V600E^ mutation (OR: 10.887, 95%CI: 1.483–79.923, *P* = 0.019) were verified to be the independent predictive factors in detecting RAIR (Table 2). In summary, the age, tumor size, pstim-Tg2/pstim-Tg1, sup-Tg2/sup-Tg1, and BRAF^V600E^ mutation could predict the RAIR. Meanwhile, only the age, pstim-Tg2/pstim-Tg1, sup-Tg2/sup-Tg1, and BRAF^V600E^ mutation were identified as independent predictive factors in detecting RAIR. This showed the peculiarly important value of quantitative Tg from a different angle.

**Table 2:** Logistic regression analyses of the baseline factors and radioactive iodine-refractory.

RAIR/non-RAIR	Univariate				Multivariate			
	*P*	OR	95%CI		*P*	OR	95%CI	
Gender (Male/Female)	0.682	0.81	0.295	2.221	.809	.794	.122	5.160
Tumor Size	**0.027**	1.439	1.042	1.988	.161	1.891	.776	4.604
Age(≥45/<45)	**0.011**	3.65	1.349	9.876	**.049**	10.484	1.009	108.942
pstim-Tg2/pstim-Tg1(>0.544/<0.544)	**<0.001**	12.745	3.322	48.897	.**041**	10.829	1.098	106.789
sup-Tg2/sup-Tg1 (>0.972/<0.972)	**<0.001**	34.125	8.174	142.458	**.004**	54.232	3.614	813.853
BRAF (+/-)	**0.006**	4.091	1.497	11.18	**.019**	10.887	1.483	79.923
AJCC T Stage	0.338	1.338	0.738	2.426	.069	.191	.032	1.134
AJCC N Stage	0.592	0.794	0.342	1.843	.177	.294	.050	1.737
Extra-thyroid extension	0.343	2.25	0.421	12.028	.269	9.381	.177	497.314
Multifocality/Unifocality	0.69	0.818	0.305	2.195	.077	.098	.007	1.286

Abbreviations: ps-Tg: pre-ablative stimulated thyroglobulin at the first (pstim-Tg1) or second (pstim-Tg2) radioactive iodine (RAI) therapy, sup-Tg: suppressed Tg before (sup-Tg1) or after (sup-Tg2) the second RAI therapy.

### Non-RAIR duration according to the Tg2/Tg1 ratio

**Fig 4:**
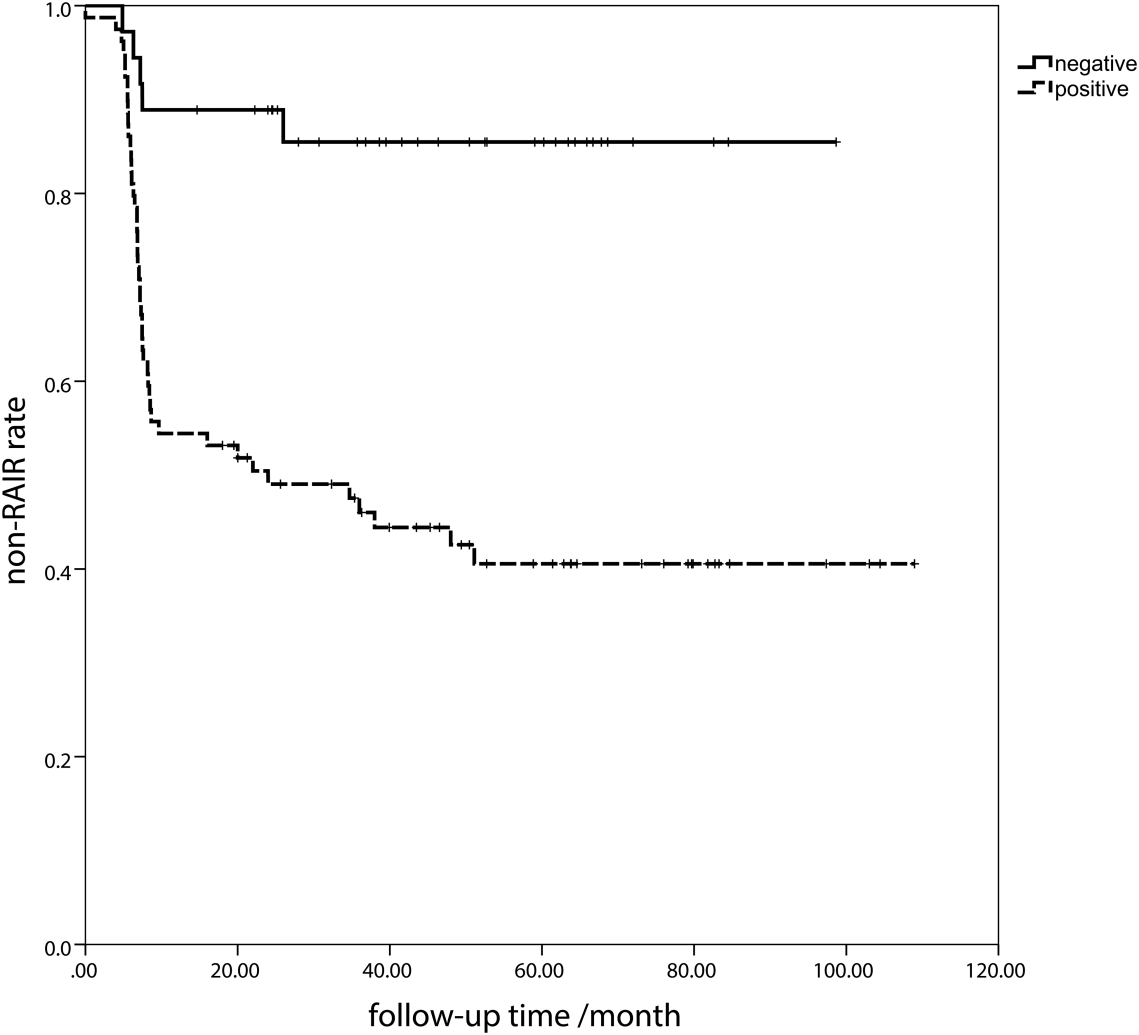
The non-radioactive iodine-refractory (RAIR) duration between groups by classification of pre-ablative stimulated Tg (pstim-Tg). The positive was defined as pstim-Tg2/pstim-Tg1≥0.544, the opposite negative.

**Fig 5:**
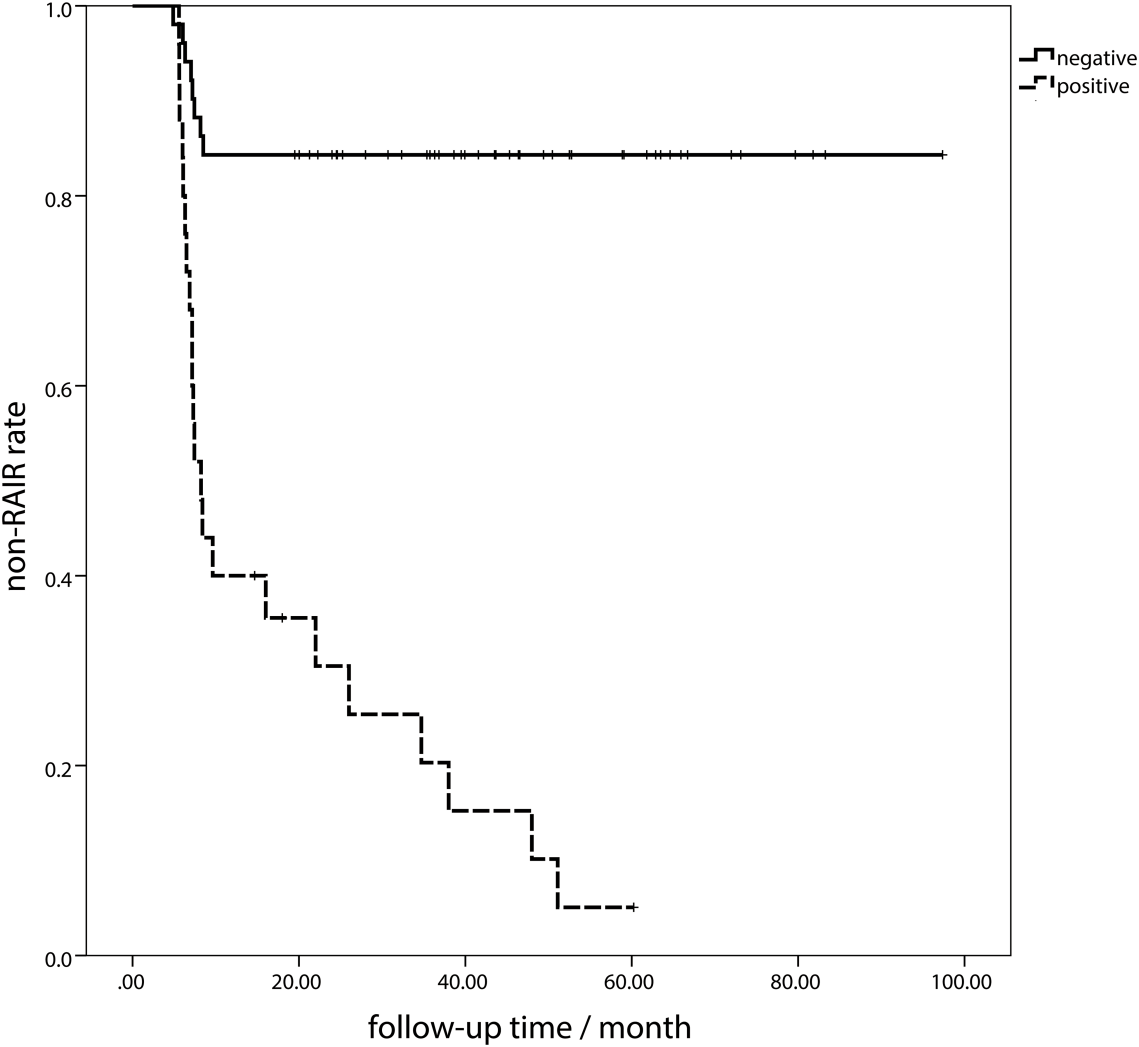
The non- radioactive iodine-refractory (RAIR) duration between groups by classification of suppressed Tg (sup-Tg). The positive was defined as sup-Tg2/sup-Tg1 ≥0.972, the opposite negative.

To further evaluate the merits of quantitative Tg in evaluating the long-term prognosis, a time to RAIR analysis was performed. Patients with positive Tg2/Tg1 were more likely refractory to RAI therapy. In this section, the presence of RAIR was defined as the end-point event, and the survival time (non-RAIR duration) was defined as the time period from the initial diagnosis of thyroid cancer to the end-point event occurrence. Patients were divided into two groups according to the pstim-Tg2/pstim-Tg1≥0.544 (Fig 4) and sup-Tg2/sup-Tg1 ≥0.972 (Fig 5). The Tg2/Tg1 ratio greater than the cut-off value (0.544 and 0.972, respectively)was deemed as positive, the opposite as negative. The medium non-RAIR survival was 20.1 months for pstim-Tg positive group and 42.6 months for negative group (*P*<0.001) with a hazard ratio (HR) of 5.390 (95% CI 2.137–13.594, *P*<0.001) (Fig 4). The medium non-RAIR duration was 8.2 months for sup-Tg positive group and 39.9 months for negative (*P*<0.001), with HR of 9.531 (95%CI 4.184–21.709, *P*<0.001) (Fig 5).

## Discussion

RAI is the mainstay for the management of distant metastatic DTC, especially for patients with pulmonary metastasis, while its efficacy peculiarly depends on the ability of radioiodine uptake in their metastatic lesions. The non-avidity in these lesions usually indicates more tendency of RAIR and less favorable outcome. Owing to the low survival rate, many researchers focused on the RAIR-DTC these years and gained impressive achievements [2,12,13,14]. The diagnosis and treatments of RAIR-DTC have been hotspots worldwide [5,15]. Till now, the diagnosis of RAIR-DTC mainly relies on WBS and trend of Tg after multiple times of RAI therapy, combining with the findings of CT, ^18^FDG-PET/CT, which is high-cost and time-consuming. As the increasing risk of adverse effect from repeated high-dose RAI and limited benefit from RAI, predictive markers regarding an earlier identification of RAIR and timely preventing such patients from unnecessary RAI therapy are highly desired. It may not only reduce the risk of progression during the thyroid hormone withdraw (THW) in some countries like China, but also gains more time for other effective therapy.

Tg, as a specific biomarker produced by the thyroid tissue and DTC lesions, has been reported to be both sensitive and convenient means in the surveillance of patients with DTC during follow-up. Furthermore, it has been confirmed as a predictor of distant metastasis, recurrence, and prognosis [2,16,17]. Due to easily obtained and low-cost, Tg has been advocated during the RAI therapy and follow-up [2]. Song et al. reported the decreasing Tg from 683 to 311 ng/mL after the RAI therapy in ^131^I-avid patients [11], suggesting the connection between the efficacy of RAI therapy and the variation of Tg level. In the 2015 ATA guideline, the Tg plays a great part in the response system predicting the clinical outcome. However, little has been reported on the relationship between the quantitative Tg analysis and RAIR-DTC. Hence, in this research, we tend to use quantitative Tg, a handy biomarker, to early and easily detect the occurrence of RAIR.

In our research, the Tg level presented its significance in two aspects. Firstly, as the Fig 1 showed, the Tg level and trend were fairly different between the RAIR and non-RAIR DTC group. This might shed light on the aggressive tumor burden reflected by the persistent high Tg level being the pejorative factor. If the Tg level does not decrease appreciably after RAI therapy, the patients may benefit little. Owing to reflecting the tumor burden [6], our results suggested that the change of Tg reflected by quantitative Tg would give evidence to the tumor response to the RAI therapy. Secondly, the response of Tg might be a good indicator in predicting the RAIR-DTC. The pstim-Tg response showed a higher sensitivity than sup-Tg (0.9 VS 0.733), whereas, the sup-Tg response possessed a higher specificity than pstim-Tg (0.935 VS 0.477). This is consistent with the higher sensitivity of pstim-Tg as previous studies reported [2,18]. Though limited by specificity, pstim-Tg seems to be an easy tool to screen the RAIR-DTC. We also attempted to use pstim-Tg/TSH to minimize the effect of TSH level, nevertheless, the pstim-Tg showed non-inferior diagnostic performance to pstim-Tg/TSH. So for the practicability of clinical use, we only take pstim-Tg for further analysis. On the other hand, the sup-Tg, considering its high specificity, is a wonderful biomarker in determining the RAIR-DTC. Consequently, we explored the predicting value of quantitative Tg response in refractory to RAI.

Furthermore, in the univariate and multivariate analysis, the age, Tg response and BRAF^V600E^ mutation were correlated to the resistance to RAI therapy. According to the AJCC TNM Stage [2], the age older than 45 was relevant to the higher death risk. Here comes to a deduction that older age presents a greater possibility to RAIR, resulting in an increased risk of mortality. In our study, BRAF^V600E^ mutation was also detected in the primary focus. It is well known that the BRAF^V600E^ mutation is related to the recurrence and poor prognosis [19,20,21]. Furthermore, our previous study demonstrated that the BRAF^V600E^ mutation was correlated to the non-^131^I-avid of DTC lesions in distant metastasis [13]. Our research comes to a similar conclusion that the BRAF^V600E^ mutation was a predictor to RAIR. In the RAIR duration analysis, the non-RAIR durations were significantly different between the positive and negative groups (Fig 3). This may suggest the efficacy of Tg2/Tg1 level in predicting the occurrence of RAIR on the other side. Patient with higher Tg2/Tg1 ratio than the cut-off values will have a greater risk to be refractory to the RAI with HRs of 5.390 and 9.531 in pstim-Tg and sup-Tg, respectively. Furthermore, less non-RAIR durations may also be revealed by the Tg response, which needs more caution.

To our knowledge, though retrospective, our study might be the first and largest work in exploring the prognosis value of quantitative Tg in RAIR. Further work with larger samples and more factors is in the process.

In conclusion, our study indicated the predicting value of quantitative Tg response in early detecting the refractory to RAI in DTC patients with pulmonary metastasis. Both quantitative pstim-Tg and sup-Tg responses were useful in forecasting the performance of RAIR-DTC. Age, BRAF^V600E^ mutation, and quantitative Tg response were independent predictors in predicting RAIR-DTC.

## Supporting information

S1 File The supporting data is the originally data of included patients in our study. In the RAIR row, 0 refers to the patients with non-radioactive iodine-refractory thyroid cancer; while 1 refers to radioactive iodine-refractory thyroid cancer. In the stimu-Tg2/Tg1 and sup-Tg2/Tg1 rows, the values refer to the Tg2/Tg1 ratio.
(XLSX)
